# Solvothermal Guided V_2_O_5_ Microspherical Nanoparticles Constructing High-Performance Aqueous Zinc-Ion Batteries

**DOI:** 10.3390/ma17071660

**Published:** 2024-04-04

**Authors:** Xianghui Jia, Kaixi Yan, Yanzhi Sun, Yongmei Chen, Yang Tang, Junqing Pan, Pingyu Wan

**Affiliations:** 1 National Fundamental Research Laboratory of New Hazardous Chemicals Assessment and Accident Analysis, Institute of Applied Electrochemistry, Beijing University of Chemical Technology, Beijing 100029, China; 2021200870@buct.edu.cn (X.J.); 2022201080@buct.edu.cn (K.Y.); chenym@mail.buct.edu.cn (Y.C.); tangyang@mail.buct.edu.cn (Y.T.); pywan@mail.buct.edu.cn (P.W.); 2 State Key Laboratory of Chemical Resource Engineering, Beijing University of Chemical Technology, Beijing 100029, China

**Keywords:** aqueous zinc-ion batteries, V_2_O_5_ microspheres, self-assembled, solvothermal method

## Abstract

Rechargeable aqueous zinc-ion batteries have attracted a lot of attention owing to their cost effectiveness and plentiful resources, but less research has been conducted on the aspect of high volumetric energy density, which is crucial to the space available for the batteries in practical applications. In this work, highly crystalline V_2_O_5_ microspheres were self-assembled from one-dimensional V_2_O_5_ nanorod structures by a template-free solvothermal method, which were used as cathode materials for zinc-ion batteries with high performance, enabling fast ion transport, outstanding cycle stability and excellent rate capability, as well as a significant increase in tap density. Specifically, the V_2_O_5_ microspheres achieve a reversible specific capacity of 414.7 mAh g^−1^ at 0.1 A g^−1^, and show a long-term cycling stability retaining 76.5% after 3000 cycles at 2 A g^−1^. This work provides an efficient route for the synthesis of three-dimensional materials with stable structures, excellent electrochemical performance and high tap density.

## 1. Introduction

Lithium-ion batteries dominate large-scale energy storage systems such as electric vehicles and power grids due to their high energy density and good cycling performance, but their limited resources and safety issues limit their development [1,2]. For the past few years, Aqueous Zinc Ion Batteries (AZIBs) have undergone continuous development owing to the advantages of cost effectiveness, high safety and eco-friendliness of AZIBs [3], and therefore have enormous development potential in the field of energy storage applications. At present, investigation of AZIBs cathode materials has focused on Prussian blue analogues, manganese-based materials, vanadium-based materials, organic materials, as well as transition metal sulphides, etc., [4,5,6,7,8]. China has abundant vanadium ore resources, while vanadium has a variety of valence states and rich chemical structure properties conducive to making vanadium-based materials with high energy storage capacity and to becoming AZIB cathode material with great application potential. Vanadium oxide materials have an open framework structure that can accommodate a large amount of Zn^2+^ for storage and can provide high energy density and specific capacity [9,10]. However, the V_2_O_5_ material has a low diffusion coefficient and conductivity of zinc ions in the process of charge and discharge, and its irreversible transformation and other problems have limited its use [11,12].

It is well known that the electrochemical performance of the battery is significantly influenced by the structure of the electrode materials, and designing V_2_O_5_ with different nanostructures is an effective method to improve its slow electrochemical kinetics and poor structural stability [13,14,15,16,17]. Compared with other low-dimensional materials, three-dimensional structures inherit the advantages of low-dimensional nanomaterials while retaining the basic units, and they have higher mechanical and cyclic stability [18,19,20]. For example, Li et al. reported V_2_O_5_-YS material with an egg yolk shell structure with holes, where the holes on the surface mitigate lattice expansion and the material is well reversible [21]. Hollow microsphere V_2_O_5_ materials with high specific surface area and fast diffusion kinetics have been prepared by Qin et al. [22]. Although significant progress has been made in the mass-specific capacity of Zn||V_2_O_5_ batteries, most studies have neglected the volume-specific capacity. In general, larger internal voids and particle gaps in the cathode material result in a low tap density, which is a key parameter affecting the volumetric energy density of the electrode [23]. Low tap density results in low specific volume capacity because the electrodes are thicker for the same mass of loaded material, resulting in a longer charge transport path [24,25]. From a practical point of view, a high loading capacity and a dense ordered structure are effective ways to achieve cathodes with high volume specific capacity, and it is necessary to develop V_2_O_5_ electrodes with high quality/volume-specific capacity without sacrificing cycle stability and multiplicity properties.

Herein, we reported V_2_O_5_ microsphere materials with high tap density, which was self-assembled from nanorods by a simple solvothermal and calcination method. No templating agent or chemical activator is added during the whole reaction process, and this synthesis method is environmentally friendly and efficient. This unique microsphere structure not only retains the mesoporous and single crystalline nature of the nanorods with an abundance of electrochemically active sites, but also avoids the high contact resistance caused by nanorod aggregation. In addition, the process of assembling nanorods into microspheres creates many pores, which can promote sufficient contact between the electrolytic solution and the material, while mitigating the lattice expansion problem during zinc storage to ensure cycle stability. The V_2_O_5_ microsphere cathodes prepared in Zn(NH_2_SO_3_)_2_ electrolytes have a high specific capacity (415 mAh g^−1^ at 0.1 A g^−1^) and long-term cycling stability (more than 3000 cycles at 2 A g^−1^). In addition, the V_2_O_5_ microspheres were self-assembled from nanorods, which not only have the advantages of nanorods in terms of shorter ion transport paths, but also significantly increased tap density due to the three-dimensional porous structure, providing a new idea for the development of cost-effective and excellent electrochemical performance AZIBs.

## 2. Experimental Section

### 2.1. Synthesis of V_2_O_5_ Microspheres

Anhydrous ethanol (C_2_H_5_OH, AR), ethylene glycol (C_2_H_6_O_2_, AR), and ammonium metavanadate (NH_4_VO_3_,99.9%) were purchased from Aladdin (Shanghai, China). All chemicals are used as received. Firstly, 350 mg of NH_4_VO_3_ was stirred vigorously with 40 mL of anhydrous ethanol at ambient temperature for 20 min; then, 10 mL of ethylene glycol was added and stirred for another 20 min. The mixture was reacted at 180 °C for 24 h in a 100 mL stainless steel autoclave. After cooling the reactor to ambient temperature, the products were washed alternately with deionized water and anhydrous ethanol more than three times and then dried overnight at 60 °C, and the precursor obtained was named as VOCH-20. Finally, it was calcined at 500 °C for 2 h at a heating rate of 2 °C min^−1^, and the product obtained was named V_2_O_5_-20. The preparation of V_2_O_5_ with different solvent ratios is the same as above, except that the glycol content in the solvent was varied to 10%, 40%, 60%, and 100%, and the synthesized precursors were designated as VOCH-10, VOCH-40, VOCH-60 and VOCH-100, and the corresponding samples were named as V_2_O_5_-10, V_2_O_5_-40, V_2_O_5_-60, and V_2_O_5_-100, respectively. The preparation of V_2_O_5_ nanoparticles was also carried out under the same conditions, using anhydrous ethanol as the solvent. The precursor and the sample were designated as VOCH-0 and V_2_O_5_-0, respectively.

### 2.2. Electrochemical Measurement

An appropriate amount of N-methyl-2-pyrrolidone was added to the mixed and ground V_2_O_5_ (70%), acetylene black (20%) and polyvinylidene fluoride (10%), and the stirred slurry was coated and pressed onto a titanium foil with about 1.1 mg cm^−2^ active materials loaded on the cathode and dried for 12 h at 60 °C. CR2016 button cells were assembled for electrochemical testing, with glass-fibre film and zinc flake as the diaphragm and anode respectively, and 3 mol L^−1^ Zn(NH_2_SO_3_)_2_ as the electrolyte. Using the constant current intermittent titration technique (GITT), the V_2_O_5_ electrode was charged and discharged in a constant current pulsed mode for 10 min at a voltage range of 0.4–1.4 V and at 100 mA g^−1^; then, the electrodes were left to stand for 1 h to allow the voltage to reach a steady state, which was repeated continuously until the discharge voltage (0.4 V) or charge voltage (1.4 V) was reached.

The materials characterization and other electrochemical measurements are shown in Supporting Information.

## 3. Results and Discussion

### 3.1. Structure and Morphology

**Figure 1:**
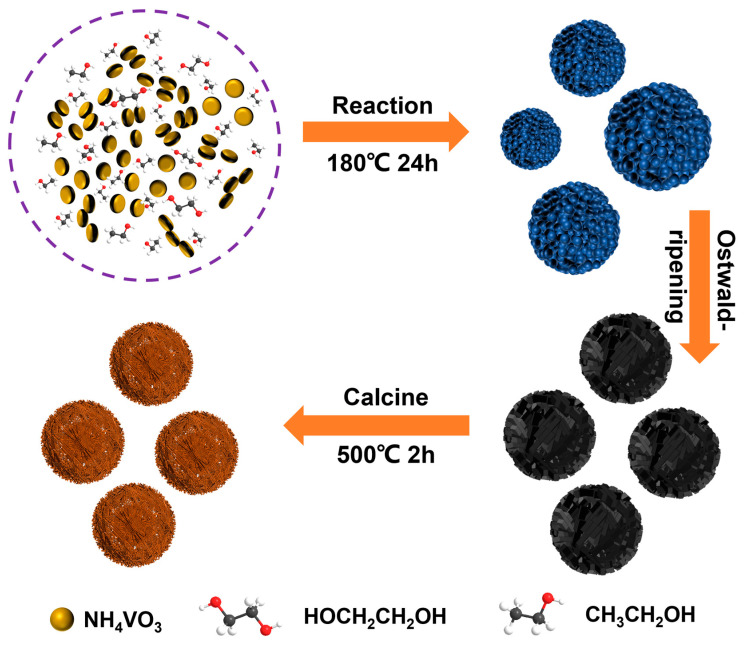
Schematic diagram of the preparation process of V_2_O_5_ microspheres.

Figure 1 shows the illustration of the synthesis process for V_2_O_5_ microspheres. At the initial stage of the solvothermal reaction, ethylene glycol is partially oxidized to acid [26], and in the weakly acidic ammonium salt solution, vanadium ions coordinate with ethylene glycol to form nanoparticles, which self-assemble into microspheres by van der Waals interaction and hydrogen bonding to reduce the surface energy. With the further reaction process, the nanorods continue to form on the surface of the microspheres, and after the Ostwald ripening process [27,28], the smaller microspheres are dissolved while the larger microspheres transform into solid microspheres of uniform size. Finally, after high-temperature annealing, the valence states of vanadium are elevated, and meanwhile, the metal- ethylene glycol complexes within the microspheres are pyrolyzed to escape gases, resulting in the formation of porous microspheres.

**Figure 2:**
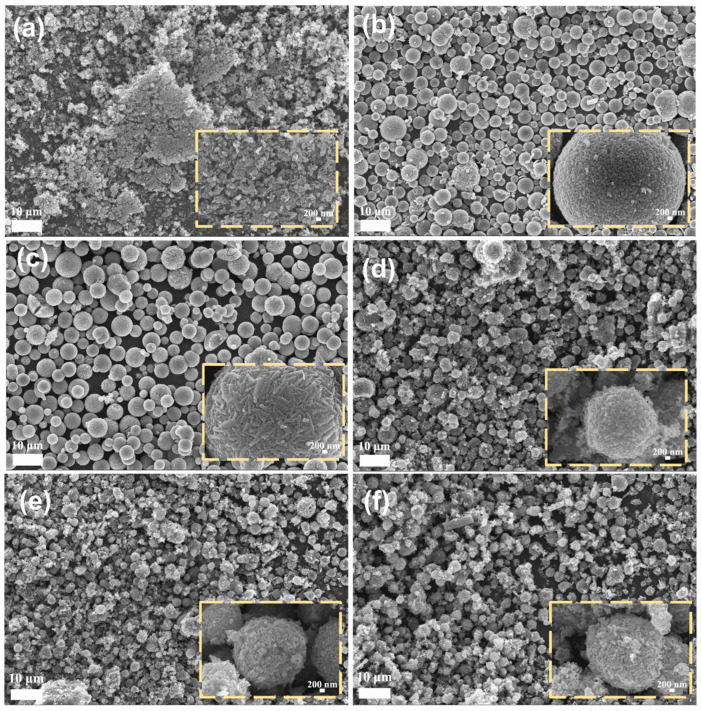
SEM images of V_2_O_5_ prepared in solvent with different volume contents of ethylene glycol: (**a**) 0%, (**b**) 10%, (**c**) 20%, (**d**) 40%, (**e**) 60%, and (**f**) 100%.

The SEM images of V_2_O_5_ prepared in a variety of solvent ratios are shown in Figure 2. From Figure 2a–c, it is clear that the V_2_O_5_ samples are transformed from nanoparticles to porous microspheres, which consist of nano-subunits that are stacked on top of each other, and the pores formed by the decomposition of the metal ethylene glycol complexes during the calcination process. The microsphere size is the largest when the volume content of ethylene glycol is 20% and the diameter of the microsphere is about 4–5 μm. Nanoparticles are not stable in the mixed solution of ethylene glycol and ethanol, so the self-assembly of nanoparticles is very fast (Figure S1), and the microsphere size becomes larger as the reaction time becomes longer.

However, Figure 2d–f shows that the microsphere structure collapses and become progressively smaller in size when the volume content of ethylene glycol is increased to 40% and above. It may be due to the high proportion of metal-ethylene glycol complexes formed in the precursors, the decomposition of the hydrocarbon and carbon-oxygen skeletons contained in these complexes during heat treatment and their escape as gases, leaving many pores. Therefore, the content of glycol in the solvent can be adjusted so that the prepared V_2_O_5_ microspheres are of moderate size and have a stable structure. It should be pointed out that the V_2_O_5_ microspheres show a significantly improved tap density compared to the V_2_O_5_ nanoparticles. After filling the measuring cylinder with the same mass of V_2_O_5_ microspheres and nanoparticles measured by tamping vibration, the volume of V_2_O_5_-20 is just 68% to that of V_2_O_5_-0 (Figure S2).

From Figure S3a, the diffraction peaks of the samples prepared in different solvent fractions coincide with the standard card of orthorhombic phase V_2_O_5_ (JCPDS No. 41-1426), and there is no impurity phase is detected, suggesting that both ethanol and ethylene glycol as the reaction solvents can provide a good environment for the material synthesis. It is noteworthy that V_2_O_5_-20 has the best crystallinity among the samples prepared in different component solvents, which is in agreement with the results of the SEM images, indicating that the content of ethylene glycol in the solvent of 20% is the optimal condition for the synthesis of V_2_O_5_, and conducive to the formation of microspheres with a porous structure. Figure S3b shows the FT-IR of the V_2_O_5_ synthesized in different solvents after calcination. The strong absorption peak at 1020 cm^−1^ is the V=O stretching vibration peak, the peak at 830 cm^−1^ is the bending vibration peak of the doubly coordinated V-O-V oxygen bond, and V-O-V asymmetric stretching vibration peak appears at 632 cm^−1^. These vibrational modes correspond to the characteristic vibrational peaks of V_2_O_5_ and are consistent with those reported in the literature [29,30].

**Figure 3:**
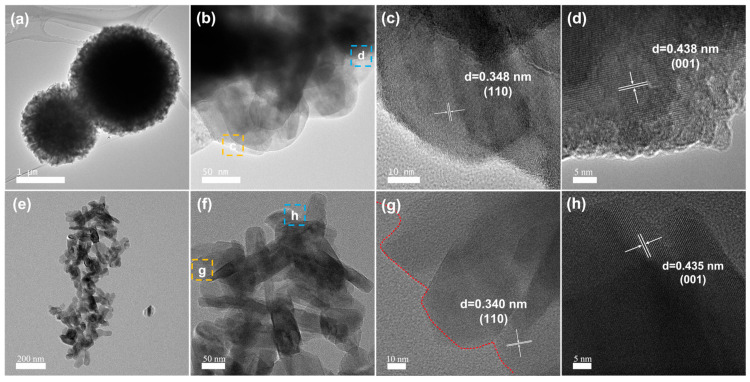
(**a**–**d**) TEM and HRTEM images of V_2_O_5_-20 microspheres, (**e**–**h**) TEM and HRTEM images of V_2_O_5_-0 nanoparticles.

In order to better reflect the influence of ethylene glycol on the structure and morphology of V_2_O_5_, we chose the V_2_O_5_-20 with the best microsphere morphology and V_2_O_5_-0 samples in the SEM as the main research objects. The uniform distribution of V and O elements on the surface of the microspheres is observed in Figure S4. From the TEM images (Figure 3a,b), it can be clearly observed that the microspheres have a solid structure, and there are many pores on the surface of the microspheres. Well-resolved lattice fringes with plane spacing of 0.348 nm and 0.438 nm correspond to the (110) and (001) crystal planes of V_2_O_5_-20, respectively (Figure 3c,d). For comparison, V_2_O_5_-0 was also characterized (Figure 3e–h), which exhibits an irregular nanoparticle morphology but essentially the same crystal structure as V_2_O_5_-20.

**Figure 4:**
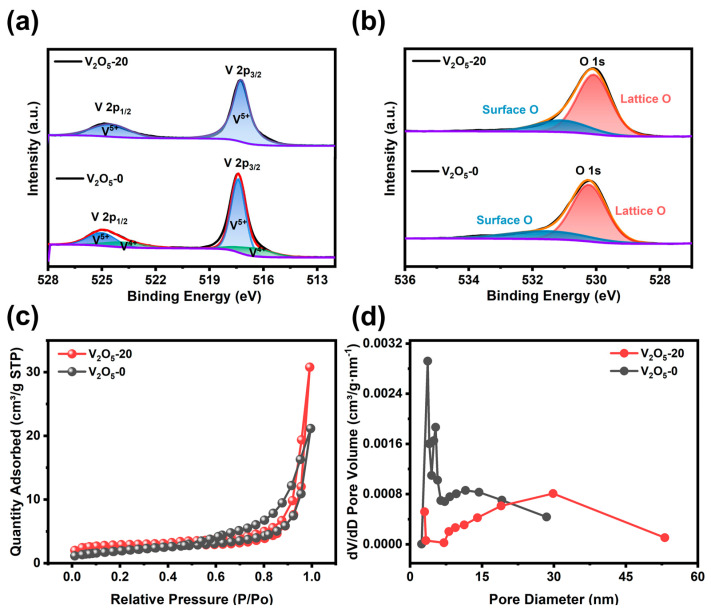
(**a**,**b**) V 2p and O 1s core-level spectrum of V_2_O_5_, (**c**,**d**) N_2_ adsorption–desorption curves and the corresponding pore size distribution curves of V_2_O_5_.

As shown in Figure S5, the XPS full spectrum of V_2_O_5_-20 only notes the V, O, and C elements. Figure 4a shows the V2p spectrum of V_2_O_5_ samples, and it can be seen that the peaks of V 2p_1/2_ and V 2p_3/2_ are located at 524.5 eV and 517.2 eV in V_2_O_5_-20, respectively [31]. The binding energy separation is about 7.3 eV, which is very consistent with the oxidation state of V^5+^. However, the binding energies of V^5+^ 2p_3/2_ (517.4 eV) and V^4+^ 2p_3/2_ (516.4 eV) indicate that some of the V in V_2_O_5_-0 is reduced to the lower valence state, which may be one of the reasons for its poor capacity. The peaks in the O 1s spectra at 530.1 eV and 531.1 eV can be attributed to lattice oxygen and surface oxygen in V_2_O_5_, respectively (Figure 4b) [32]. The porous properties of V_2_O_5_ materials were investigated by N_2_ adsorption–desorption measurements (Figure 4c,d). From the IUPAC classification, the adsorption isotherms of both V_2_O_5_-20 and V_2_O_5_-0 are part of the II- type isotherm in the H3-type hysteresis loop, indicating the mesoporous properties of the material. The porous V_2_O_5_-20 microsphere displays the specific surface area of 9.40 m² g^−1^ and the pore size distribution in the range of 20–40 nm, and its shows higher pore volume of 0.047 cm^3^ g^−1^ compared to nanoparticulate V_2_O_5_-0 (Table S1). In conclusion, the microspheres with high specific surface area have many pores, which is beneficial to increasing the contact area between the electrolytic solution and the material [33,34]. In addition, due to the aggregation of nanorods, the diffusion path of Zn^2+^ is shortened, which is expected to enhance electrochemical kinetics.

### 3.2. Mechanistic Analysis of Microsphere Formation

**Figure 5:**
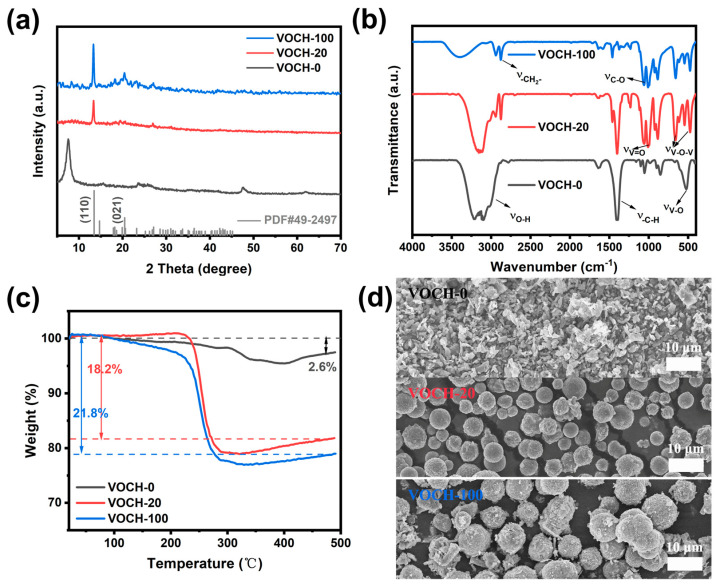
(**a**) XRD patterns, (**b**) FT-IR spectra, (**c**) TG curves and (**d**) SEM images of the three VOCH precursors.

The structure of ethylene glycol is HOCH_2_-CH_2_OH, which is a typical double-dentate ligand that can easily coordinate with the central metal ion in solvothermal reactions. Therefore, it is often used as a structure-directing agent for the preparation of metal oxides in some interesting forms [35,36,37,38]. In this work, ethylene glycol was used as a structure-directing agent, and ethanol was miscible for a solvothermal reaction to prepare V_2_O_5_ microspheres. The morphology and structure of V_2_O_5_ microspheres are greatly influenced by the composition of the VOCH precursors. The XRD spectrum of VOCH-20 and VOCH-100 in Figure 5a shows a strong diffraction peak at 13°, which is consistent with the standard card of VO(CH_2_O)_2_ (JCPDS No. 49-2497). And, through literature research, we have found that many metal-ethylene glycol complexes have a distinct diffraction peak here [39,40]. Therefore, we believe that the VOCH-20 and VOCH-100 precursor is a compound formed by the coordination of ethylene glycol and vanadium ions, and the crystal structures of the VOCH precursors prepared in different solvent fractions are similar (Figure S6a), indicating that the vanadium ions are preferentially coordinated with ethylene glycol to form complexes in solvents containing ethylene glycol. However, VOCH-0 has a strong diffracted broad peak of around 7.5°, which should be a complex formed by ethanol and vanadium ions. Ethylene glycol has a strong coordination ability due to the presence of two hydroxyl groups as bidentate ligands, and the surface of the complex formed contains a large number of hydroxyl groups and other functional groups; the internal part of the material also consists of a hydrocarbon skeleton.

From Figure 5b, it is observed that the infrared spectrum of the VOCH precursors show vibrational peaks of the hydrocarbon bond at about 1400 cm^−1^ and multiple vibrational peaks of carbon-oxygen bond at around 1060 cm^−1^, which are generated by the hydroxyl group left over from the coordination process of alcohols and the vibration of the carbon–hydrogen skeleton, indicating that a large amount of organic functional groups is present on the surface of the precursor. However, only when the solvent component contains ethylene glycol can the vibrational peaks of methylene in the infrared spectrum be found at around 2870 cm^−1^, and do V-O-V symmetric and asymmetric stretching vibrational peaks appear at 478 cm^−1^ and 650 cm^−1^, which indirectly proves that the molecular formula of the ethylene glycol complexes contained in the precursors with a microsphere structure could be VO(CH_2_O)_2_. It should also be pointed out that the -OH vibrational peaks of the VOCH precursor are significant and shift to higher wavenumbers as the content of ethylene glycol in the solvent increases (Figure S6b), proving that the higher the glycol content in the solvent, the higher the proportion of glycol complexes in precursors.

Figure 5c shows the TG curves of the samples before calcination, and the results indicate that the optimal annealing temperature under the air atmosphere for the precursors is 500 °C. It can be clearly seen that the VOCH-20 precursor has a greater mass loss after calcination compared to VOCH-0, suggesting the metal-ethylene glycol complex in VOCH-20 has a higher molecular weight, and more gases are released during the pyrolysis process, thus making the structure porous, which is consistent with the SEM results. The VOCH-20 consists of microspheres with diameters of approximately 4–5 μm and an irregular sheet-like stacking structure on the surface (Figure 5d). In contrast, the VOCH-0 exhibits uniform nanoparticles. This is related to the bidentate ligand structure of ethylene glycol, which can easily connect nanoparticles in the presence of hydrogen and coordination bonds to form three-dimensional structures (Figure S7), whereas ethanol is not only a monodentate ligand, but also has a lower boiling point, which cannot provide a relatively stable environment to facilitate the assembly of nanoparticles into spheres. It is noteworthy that there are two distinct weight reductions in the TG curves of the VOCH-0 precursor: the exothermic peak at 200 °C corresponds to the removal of water of crystallization from the sample, and the exothermic peak at 385 °C is the oxidation reaction that occurs as a result of the decomposition of the complex formed by ethanol and vanadium ions (Figure S8). In contrast, the VOCH-20 and VOCH-100 precursors have only a sharp exothermic peak at 256 °C, corresponding to the decomposition and oxidation of the complexes of vanadium ions and ethylene glycol, which laterally confirms that the two precursors are similar in structure, consistent with the XRD results. In addition, the mass increase at 300–500 °C is due to the recrystallization of the sample and the oxidation of low-valence vanadium to raise the valence state.

In summary, the reaction mechanism proposed for solvents containing ethylene glycol is as follows. Firstly, under the high temperature and pressure of the solvothermal reaction, part of the glycol is oxidized to glycolic acid (Equation (2)), forming H^+^ and free VO_3_^−^ in solution, which are bound to the glycol molecule by hydrogen bonding and van der Waals forces, and then the VO_3_^-^ ions are reduced and bound to glycol to form a complex of VO(CH_2_O)_2_ (Equation (3)). Finally, the oxidative decomposition of VO(CH_2_O)_2_ in an air atmosphere produces the V_2_O_5_ product (Equation (4) and the Figure S9).

NH_4_VO_3_ → VO_3_^−^ + NH_4_^+^ (1)

2HOCH_2_CH_2_OH + 3O_2_ → HOOCCOOH + 2H_2_O ↔ HOOCCOO^−^ + H^+^ + 2H_2_O (2)

2VO_3_^−^ + 2H^+^ + 3HOCH_2_CH_2_OH → 2VO(CH_2_O)_2_ + HOCH_2_CHO + 4H_2_O (3)

4VO(CH_2_O)_2_ + 11O_2_ → 2V_2_O_5_ + 8CO_2_ + 8H_2_O (4)

### 3.3. Electrochemical Performance

#### 3.3.1. Influence of Electrode Materials on Zinc-Ion Batteries

**Figure 6:**
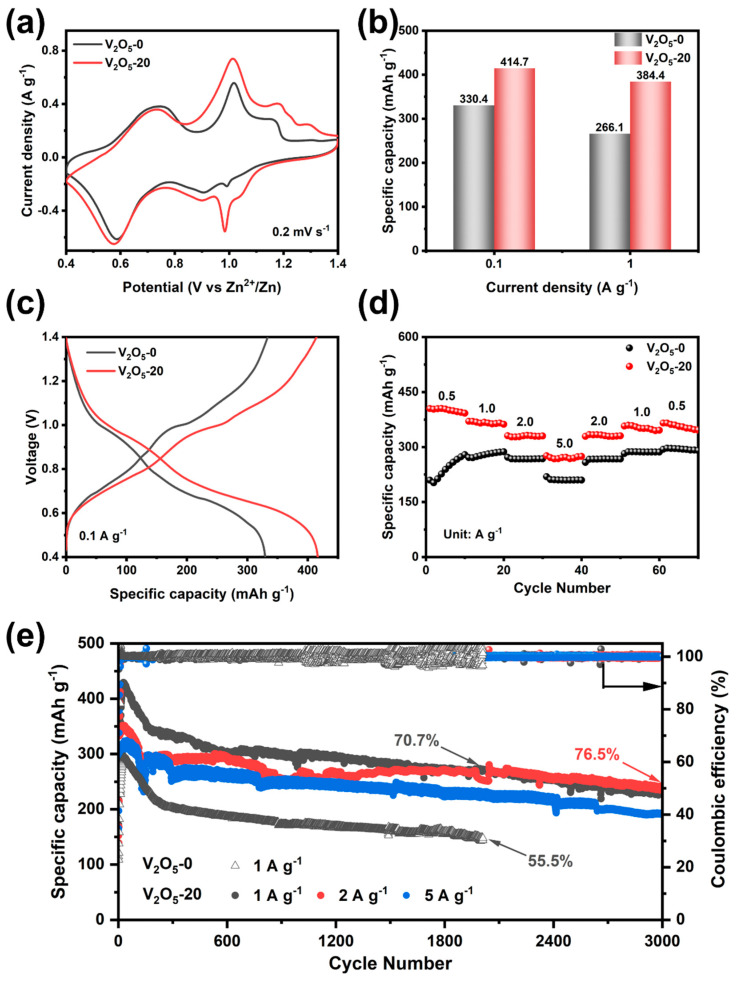
(**a**) CV curves at 0.2 mV s^−1^, (**b**) specific capacities at 0.1 A g^−1^ and 1 A g^−1^ after 15 cycles, (**c**) GCD curves at 0.1 A g^−1^ after 15 cycles, (**d**) rate performance at different current densities, (**e**) long-term cycling performance.

The electrochemical performance of V_2_O_5_ microsphere cathodes were evaluated in CR2016 button-type batteries using zinc foil as a positive electrode and 3 mol L^−1^ Zn(NH_2_SO_3_)_2_ as an electrolyte. The CV curves of the V_2_O_5_-0 and V_2_O_5_-20 electrode at a voltage range of 0.4–1.4 V are shown in Figure 6a. It is observed that the CV curves of both electrodes exhibit similar shape and show two pairs of oxidation–reduction peaks at 0.59/0.73 V and 0.99/1.02 V, corresponding to the multi-step intercalation/de-intercalation of Zn^2+^. The electrochemical properties of V_2_O_5_ microspheres synthesized under different solvent fractions were compared in Figure S10, and the results show that V_2_O_5_-20 has the best performance. As show in Figure S10a, the initial capacity of the two materials is similar, about 201 mAh g^−1^ at 0.1 A g^−1^, and the specific capacity of V_2_O_5_-20 increases to 414.7 mAh g^−1^ after 15 cycles, 84.3 mAh g^−1^ higher than that of V_2_O_5_-0 (Figure 6b). Even at high current density of 1A g^−1^, V_2_O_5_-20 achieves a high capacity of 384.4 mAh g^−1^, which is 118.3 mAh g^−1^ higher than V_2_O_5_-0. Figure 6c shows the discharge curve of the samples at 0.1 A g^−1^ after 15 cycles. It is observed that the average discharge voltage of V_2_O_5_-20 is higher than that of V_2_O_5_-0, indicating V_2_O_5_-20 may have higher energy density.

Figure 6d and Figure S11 show that the rate capability of the V_2_O_5_-20 cathode is more superior than that of V_2_O_5_-0, and the specific capacity can still reach 272 mAh g^−1^ at 5 A g^−1^, indicating that the V_2_O_5_-20 cathode remains stable at high current densities, which is inseparable from the three-dimensional structure’s superior stability. Figure 6e shows that at the specific capacity of the V_2_O_5_-20 cathode after 3000 cycles at 1, 2 and 5 A g^−1^, it can be seen that the V_2_O_5_-20 exhibits high capacity at high current densities. For instance, after 3000 cycles at 2 A g^−1^, the reversible capacity of V_2_O_5_-20 is 257 mAh g^−1^ with a capacity retention of around 76.5% and a coulombic efficiency close to 100%. At the current density of 1 A g^−1^, the specific capacity of the V_2_O_5_-0 cathode is only 148 mAh g^−1^ after 2000 cycles, and the capacity retention is only 55.5%, while V_2_O_5_-20 has higher capacity retention of 70.7%.

The higher specific capacity is attributed to the fact that the V_2_O_5_-20 cathode is essentially in the V^5+^ valence state, with a higher charge transfer number. In addition, the porous structure of the microspheres allows for a larger effective contact area with the electrolyte, providing more active sites for electrochemical reactions. Moreover, the electrochemical performance of the V_2_O_5_ microsphere electrode is superior to some reports, as shown in Table S2 [41,42,43].

#### 3.3.2. The Reaction Kinetics of V_2_O_5_ Microspheres

**Figure 7:**
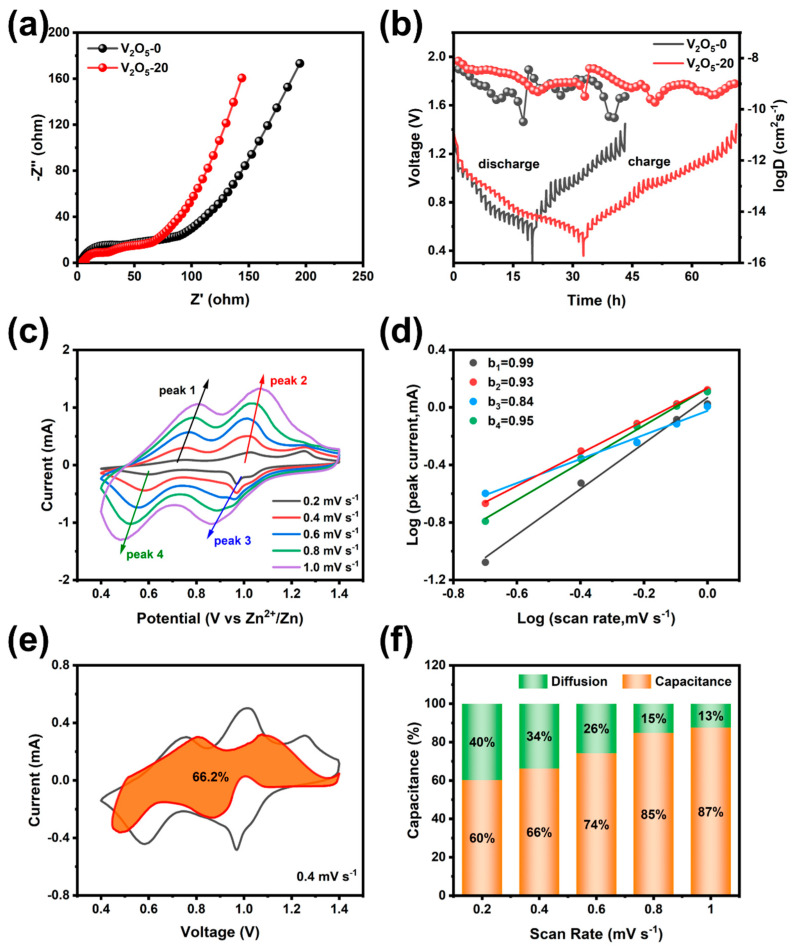
(**a**) Impedance plots of V_2_O_5_-0 and V_2_O_5_-20, (**b**) GITT curves and the corresponding Zn^2+^ diffusion coefficient of V_2_O_5_-0 and V_2_O_5_-20 in the discharge and charge process, (**c**) CV curves of the V_2_O_5_-20 electrode at diverse scan rates, (**d**) plots of log i and log ν at specific peaks current, (**e**) the capacitive contribution at 0.4 mV s^−1^ and (**f**) the capacitive contribution ratios of V_2_O_5_-20 at various scan rates.

For the purpose of understanding the zinc ion storage behavior of the V_2_O_5_-20 cathode, a detailed study of the electrochemical reaction kinetics has been carried out. As shown in Figure 7a, the impedance of the Zn//V_2_O_5_ battery was tested after 100 cycles at 1 A g^−1^. The two semicircles in the EIS spectrum can match the positive and negative interface regions of the battery. The charge transfer resistance (Rct) of the V_2_O_5_-20 cathode (65.2 Ω) is lower than that of the V_2_O_5_-0 cathode (91.0 Ω), indicating its superior conductivity. It is worth noting that in the low frequency range, V_2_O_5_-20 shows a higher slope than V_2_O_5_-0, indicating that the transfer of Zn^2+^ is faster in V_2_O_5_-20. In addition, the diffusion coefficient of Zn^2+^ (*D_Zn_*) between the electrode during cycling was implemented using the GITT. It follows that the zinc ion diffusion rate for V_2_O_5_-20 is in the order of 10^−8^ to 10^−9^ cm^2^ s^−1^, which is an order of magnitude higher than that of V_2_O_5_-0 (Figure 7b). This suggests that the construction of three-dimensional structured V_2_O_5_ microspheres is beneficial for improving the reaction kinetics of the cathode.

To gain a deep insight into the outstanding rate performance of the V_2_O_5_ porous microspheres electrode, the diffusion behavior of Zn^2+^ in electrode materials was studied by CV measurements at a series of scan rates from 0.2 to 1.0 mV s^−1^. Figure 7c shows that with increasing scan speed, all CV curves maintain a similar shape, showing the good reversibility of the electrodes. The shift in the redox peaks is due to electrode polarization at larger scan rates. The relationship between the sweep rate and the response peak current on the CV curves is determined by Equation (5):

*i* = *aν^b^* (5)

Generally speaking, a *b* value of 0.5 suggests the electrochemical reaction is controlled by diffusion, while a *b* value of 1.0 represents surface capacitive behavior, and the b is generally taken in the range of 0.5 and 1. To facilitate the calculation of the value of b, Equation (5) can also be expressed by Equation (6):

*log*(*i*) = *b log*(*ν*) + *log*(*a*) (6)

It can be seen in Figure 7d that the *b* values on the slopes of the four redox peaks are 0.99, 0.93, 0.84, and 0.95, respectively, revealing that the charge storage mode of the V_2_O_5_-20 electrode is mostly controlled by surface capacitance. In addition, at a certain scan rate, the cell capacity contribution can be categorized into capacitance-controlled (*k*_1_*ν*) and diffusion-controlled (*k*_2_*ν*^1/2^) reactions. Equation (6) can also be written as:

*i* = *k*_1_*ν* + *k*_2_*ν*^1/2^ (7)

As shown in Figure 7e, the contribution ratio at 0.4 mV s^−1^ is 66.2% of the total charge. As the scanning rate rises (Figure 7f), surface capacitance increases from 60% to 87%; the high capacitance contribution rate is one of the reasons for the high rate performance of V_2_O_5_-20 electrodes in terms of total capacity, which has been discussed in previous literature on cathode materials for zinc-ion or lithium-ion batteries [44,45].

#### 3.3.3. Investigation of the Energy Storage Mechanism of V_2_O_5_ Microspheres

In order to investigate the morphology or structural changes of V_2_O_5_ microspheres used as cathodes in AZIBs during the charge/discharge process, the batteries were removed at the end of the charge state and disassembled for testing. It can be clearly observed from Figure S12 that the color of the battery separator deepens with the number of cycles, indicating that the residual active material on the battery membrane increases. At the same time, the phase transitions and reaction mechanisms occurring at the electrodes during cycling were further explored using ex situ SEM and EDS tests.

**Figure 8:**
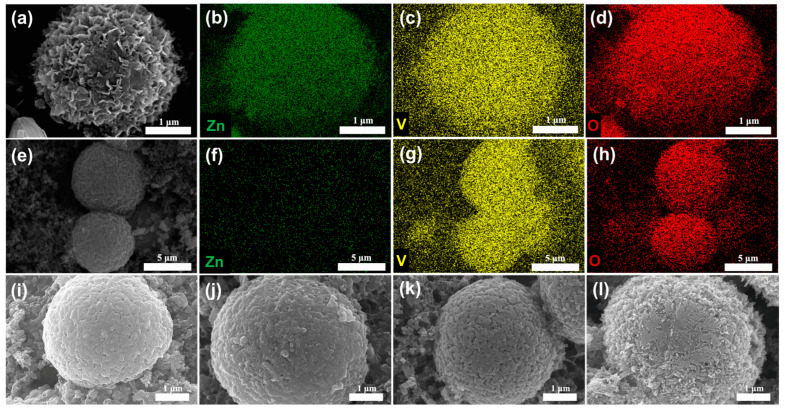
Elemental mapping images of V_2_O_5_-20 electrode (**a**–**d**) discharged to 0.4 V, (**e**–**h**) charged to 1.4 V and SEM images of V_2_O_5_-20 electrode at (**i**) initial state, (**j**) 10th charged to 1.4 V, (**k**) 100th charged to 1.4 V, (**l**) 500th charged to 1.4 V.

Figure 8a–h shows that Zn, V, and O elements are uniformly distributed in the electrodes after discharge, and the nanorods structure on the microsphere surface disappears during the discharge process, which is probably due to the formation of a new physical phase. After the charging process, the microsphere structure is recovered, which confirms that the intercalation reaction of Zn^2+^ is reversible. As shown in Figure 8i–l, SEM images can reflect the electrode morphology changes during the cycling process. It can be observed that after 100 cycles, the surface of V_2_O_5_-20 does not change significantly and maintains a smooth and porous microsphere structure. It is noteworthy that the V_2_O_5_ electrode still retains a good microsphere structure after 500 cycles, but the surface of the microspheres become rougher. This may be due to the irreversible change of the lattice spacing of the V_2_O_5_ microspheres by multiple intercalation/deintercalation of Zn^2+^ during the charging/discharge process, resulting in the change in the surface morphology.

**Figure 9:**
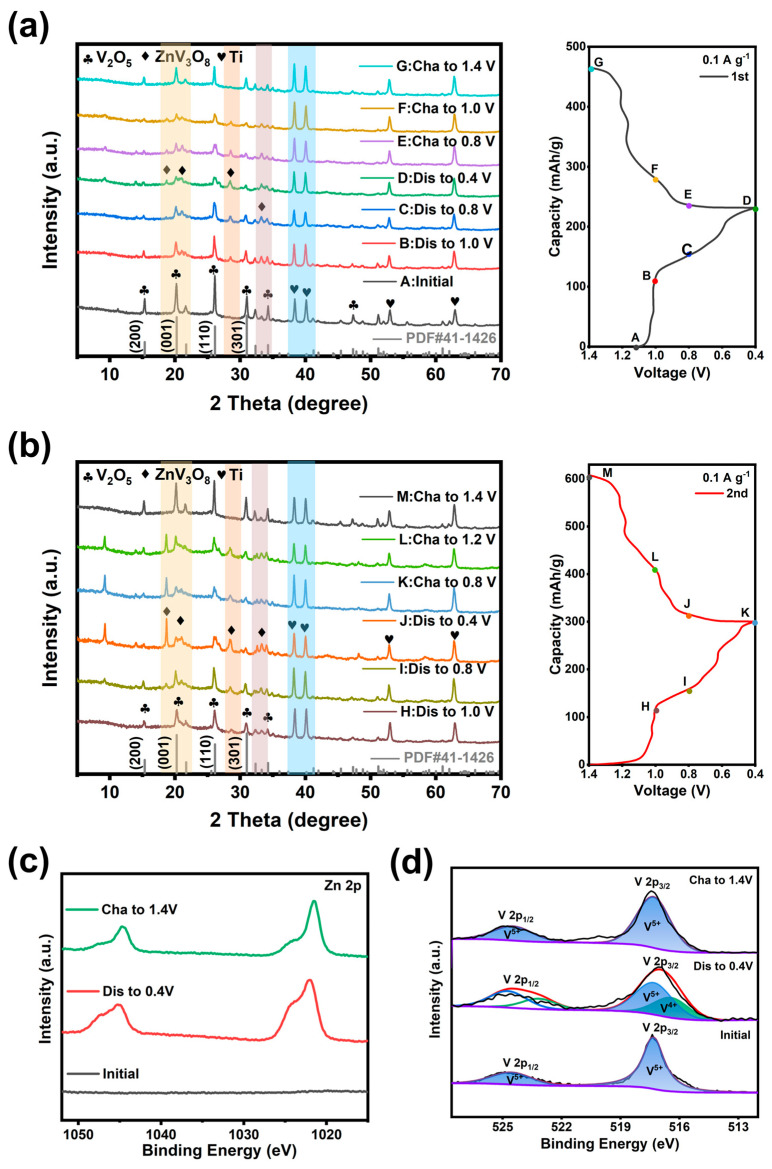
Ex-situ XRD patterns of V_2_O_5_-20 electrode at various charge/discharge states. (**a**) First cycle, (**b**) second cycle and XPS spectra after charging at 1.4 V, discharging at 0.4 V and initial potential of (**c**) Zn 2p, (**d**) V 2p.

To further demonstrate the charge storage mechanism in V_2_O_5_ microspheres, the crystal phase transitions of V_2_O_5_-20 electrodes under different charging and discharging states were detected by ex situ XRD, and the patterns in the first two cycles are show in Figure 9a,b. Figure 9a indicates only the peaks of V_2_O_5_, and Ti foil (JCPDS No. 44-1294) appear in the initial phase for the first cycle. With the decrease in discharged voltage, the original (200), (001), (110) and (301) planes of V_2_O_5_ are gradually weakened or even disappear because Zn^2+^ is gradually intercalated into the cathode material. Meanwhile, new diffraction peaks appear at 18.78° and 28.70°, indicating the formation of a new phase of ZnV_3_O_8_ (JCPDS No. 24-1481) [46]. During the charge process, Zn^2+^ moves from the positive to the negative electrode, and the characteristic peaks of V_2_O_5_ are gradually enhanced again, meaning the cathode gradually returns to the initial state.

A similar situation persists in the second cycle (Figure 9b), where the (300) and (411) crystalline planes of ZnV_3_O_8_ gradually appear as the voltage decreases during the discharging process, and the charging process is the opposite. Notably, the XRD pattern of the electrode charged to 1.4 V for the second time is the same as that for the first time, indicating that the V_2_O_5_ microspheres have good structural reversibility in the Zn(NH_2_SO_3_)_2_ electrolyte. In addition, the HRTEM images in Figure S13 can also reflect the structural change during the charging/discharging process. With the intercalation of Zn^2+^, the interplanar spacing of the (001) plane of V_2_O_5_ becomes larger and transforms into the (300) plane of ZnV_3_O_8_, and as the charging process continues, the d values of the (001) plane almost recover to the original state, confirming that Zn^2+^ are successfully migrated into the host structure of V_2_O_5_.

The changes in elemental states of V_2_O_5_-20 electrodes during the insertion and extraction process were further investigated using XPS. As shown in Figure 9c, the existence of Zn 2p_3/2_ and Zn 2p_1/2_ diffraction peaks confirm the intercalation of Zn^2+^ into the V_2_O_5_-20 electrode during the charging state. In contrast, the XPS spectrum of Zn showed unchanged peak shape or position after charging, indicating that the oxidation state of Zn did not change. As shown in Figure 9d, when the electrode was discharged to 0.4 V, a pair of diffraction peaks appeared at 516.3 eV (V 2p_3/2_) and 523.2 eV (V 2p_3/2_), proving that charge transfer occurred during the intercalation process of Zn^2+^, and V^5+^ was partially reduced to V^4+^. In the charging state, the V^4+^ signal peak decrease, while the V^5+^ peaks return to initial state, demonstrating that Zn^2+^ can reversibly extraction/insertion into/from V_2_O_5_ microspheres.

## 4. Conclusions

In this paper, self-assembled V_2_O_5_ microspheres were successfully synthesized by adjusting the reaction solvent components without adding any template. The V_2_O_5_ microsphere electrode displays a high reversible capacity of 414.7 mAh g^−1^ at 0.1 A g^−1^ and 76.5% capacity retention after 3000 cycles at 2 A g^−1^, indicating good cycling stability. The excellent Zn^2+^ storage performance of the V_2_O_5_ microspheres can be attributed to the higher chemical diffusion coefficients of Zn^2+^ compared to that in V_2_O_5_ nanoparticles and the unique porous structure of microspheres, which can promote the reversible embedding of Zn^2+^ and buffer the lattice expansion to keep the electrode structure stable after many cycles. Moreover, compared with nanoparticles, the V_2_O_5_ microspheres exhibit significantly increased tap density, which will help improve the energy density of the battery. This work explores a simple and feasible way for synthesizing a cathode with three-dimensional structures for AZIBs.

## Data Availability

Data are contained within the article and Supplementary Materials.

## Supplementary Materials

The following supporting information can be downloaded at: https://www.mdpi.com/article/10.3390/ma17071660/s1, Figure S1: Size of the V_2_O_5_-20 microspheres at different reaction times (from 6 h to 24 h); Figure S2: Volume comparison of (a) V_2_O_5_-0, (b) V_2_O_5_-20, and (c) V_2_O_5_-100 with the same mass (5 g); Figure S3: XRD patterns (a) and FT-IR spectra (b) of V_2_O_5_ samples; Figure S4: Elemental distribution of V_2_O_5_-20; Figure S5: XPS survey spectra of (a) V_2_O_5_-0, (b) V_2_O_5_-20; Figure S6: XRD patterns (a) and FT-IR spectra (b) of VOCH precursors; Figure S7: Schematic illustration of the chaining of ethylene glycol or ethanol with nanoparticles; Figure S8: TGA/DSC profiles of the three V_2_O_5_ precursors before calcination; Figure S9: Schematic of V_2_O_5_ formation by calcination of VOCH precursors; Figure S10: Cycle performance of different V_2_O_5_ electrodes at (a) 0.1 A g^−1^, (b) 1 A g^−1^; Figure S11: Specific capacity of V_2_O_5_-20 cathode at various current densities; Figure S12: The pictures of battery separators after different recycle times: (a) 1st, (b) 10th, (c) 100th and (d) 500th; Figure S13: HRTEM images of V_2_O_5_-20 electrode (a) discharged to 0.4 V, (b) charged to 1.4 V; Table S1: The BET surface area, pore volume and average pore size of V_2_O_5_-0 nanoparticles and V_2_O_5_-0 microspheres samples; Table S2: A survey of V_2_O_5_-based electrode materials with three-dimensional structures for AZIBs.
