# Supplementary material for: Solvothermal Guided V2O5 Microspherical Nanoparticles Constructing High-Performance Aqueous Zinc-Ion Batteries
Source: Materials (Basel). 2024 Apr 4;17(7):1660. doi: 10.3390/ma17071660 (PMC11012685; doi:10.3390/ma17071660)
Supplement: Supplementary file 1 [file materials-17-01660-s001.zip › materials-2940263-supplementary.pdf]

Supporting Information

Solvothermal guided V_2O_5 microspherical nanoparticles constructing high performance aqueous zinc-ion batteries

Xiangnhui Jia†, Kaixi Yan†, Yanzhi Sun†,*, Yongmei Chen†, Yang Tang†, Junqing Pan‡,* and Pingyu Wan†

†National Fundamental Research Laboratory of New Hazardous Chemicals Assessment and Accident Analysis, Institute of Applied Electrochemistry, Beijing University of Chemical Technology, Beijing 100029, China.
‡State Key Laboratory of Chemical Resource Engineering, Beijing University of Chemical Technology, Beijing 100029, China.
*E-mails: sunyz@buct.edu.cn (Y. Sun); jqpan@buct.edu.cn (J. Pan). Tel./Fax: 8610-64435452.

Experimental Section

Materials characterization

The crystal structure of the V_2O_5 samples were analyzed by X-ray diffraction (XRD) and the morphology and elemental content of V_2O_5 was investigated by transmission electron microscopy (TEM), scanning electron microscopy (SEM) and X-ray energy spectrometry (EDS). X-ray photoelectron spectroscopy (XPS) was carried out with Thermo Scientific K-Alpha to characterize the surface chemistry of the electrode materials and the valence state of vanadium. Fourier transform infrared spectroscopy (FT-IR) was used to test the chemical bonding and molecular structure of the VOCH precursors and V_2O_5 samples. The composition and decomposition temperature of the ethanol or glycol complexes in the VOCH precursor were studied by thermogravimetric analysis (TGA/DSC) in an air atmosphere in the temperature range 0~500 °C at a heating rate of 10 °C min^{-1}. The specific surface area was determined using a fully automated specific surface area and porosity analyzer according to the Brunner-Emmett-Taylor (BET) method.

Electrochemical measurement

Charge-discharge cycles of the cells were tested at room temperature using a LAND-CT3002A multi-channel battery test system. Cyclic voltammetry (CV) and electrochemical impedance spectroscopy (EIS) tests were carried out on Zn||V_2O_5 cells using an electrochemical workstation (CHI 660E) with scanning voltage from 0.4 to 1.4 V and AC impedance test at open circuit voltage.

The diffusion coefficient of Zn^{2+} (D_{Zn}) was calculated according to the following equation:

$$D_{Zn}=\frac{4L^2}{\pi\tau}\left(\frac{\Delta E_s}{\Delta E_t}\right)^2 \tag{S1}$$

In Equation (S1), τ is the pulse duration of the constant current, L is the Zn^{2+} diffusion path, ΔE_s is the voltage change of the termination voltage of two adjacent relaxation steps and ΔE_t is the voltage difference during the current pulse, subtracting the IR drop.

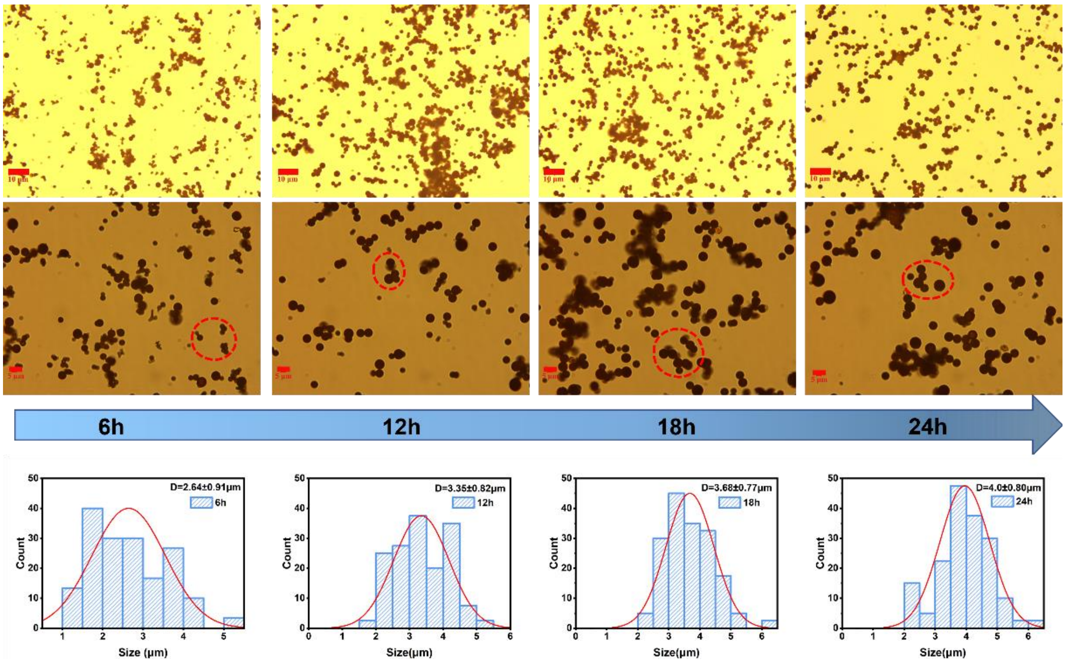

Figure S1. Size of the V_2O_5-20 microspheres at different reaction times (from 6 h to 24 h).

It can be seen for Figure S1 that the microspheres became larger and more uniform in size as the reaction time increased. The average diameter of the synthesized microspheres was 4 μm at the maximum reaction time of 24 h.

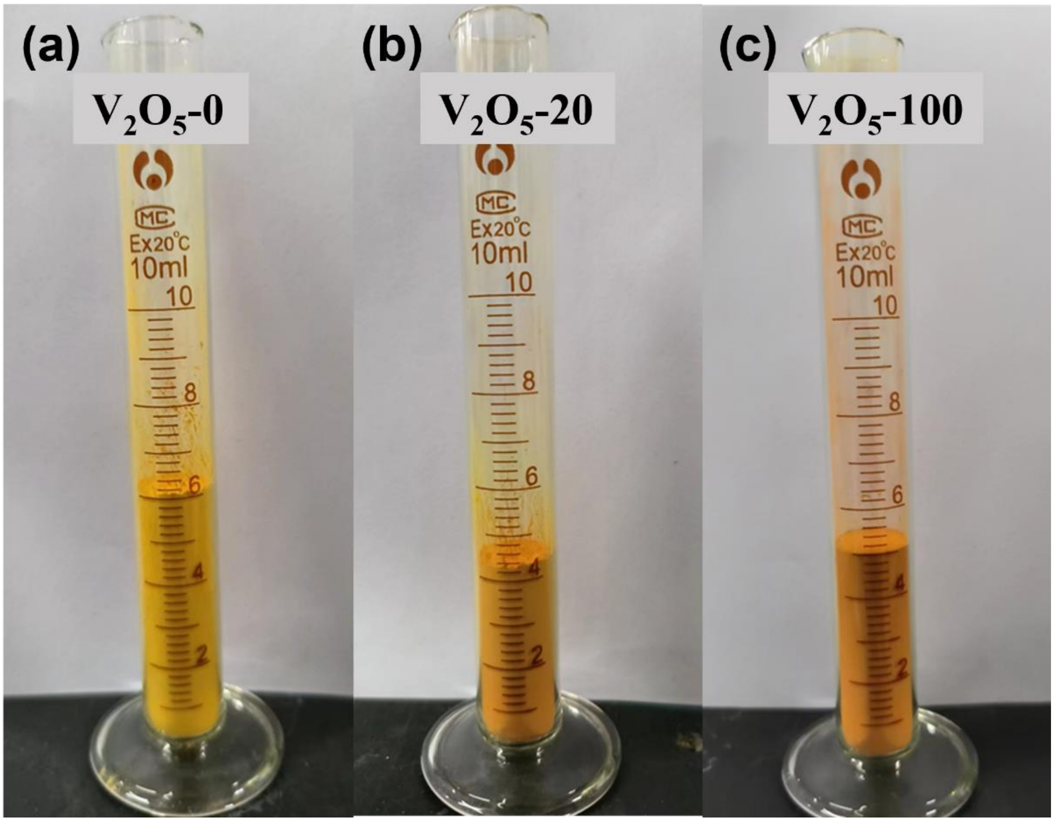

Figure S2. Volume comparison of (a) V_2O_5-0, (b) V_2O_5-20, and (c) V_2O_5-100 with the same mass (5 g).

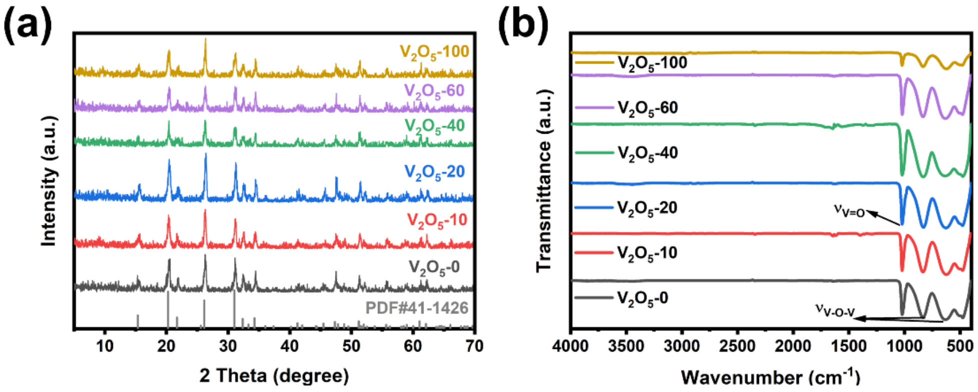

Figure S3. XRD patterns (a) and FT-IR spectra (b) of V_2O_5 samples.

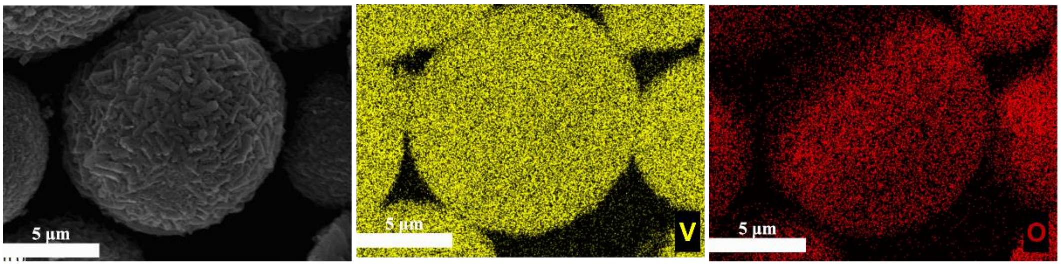

Figure S4. Elemental distribution of V_2O_5-20.

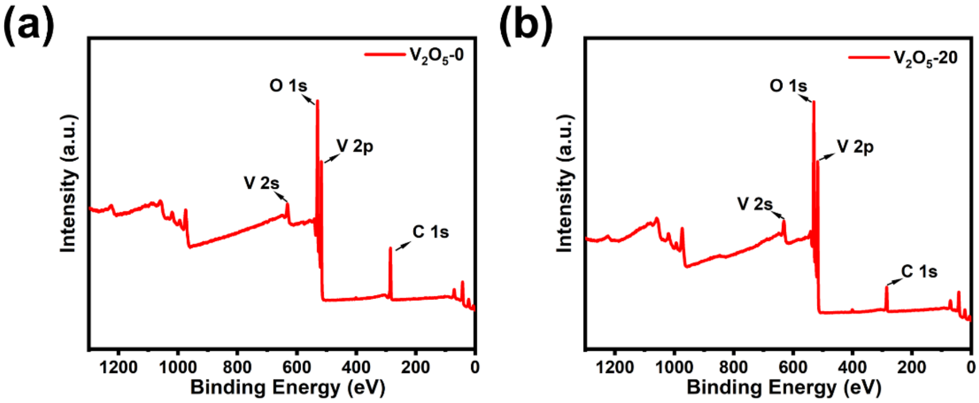

Figure S5. XPS survey spectra of (a) V_2O_5-0, (b) V_2O_5-20.

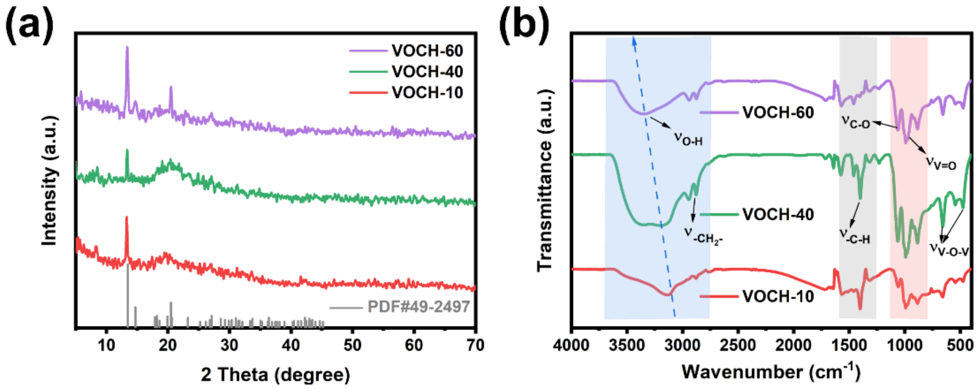

Figure S6. XRD patterns (a) and FT-IR spectra (b) of VOCH precursors.

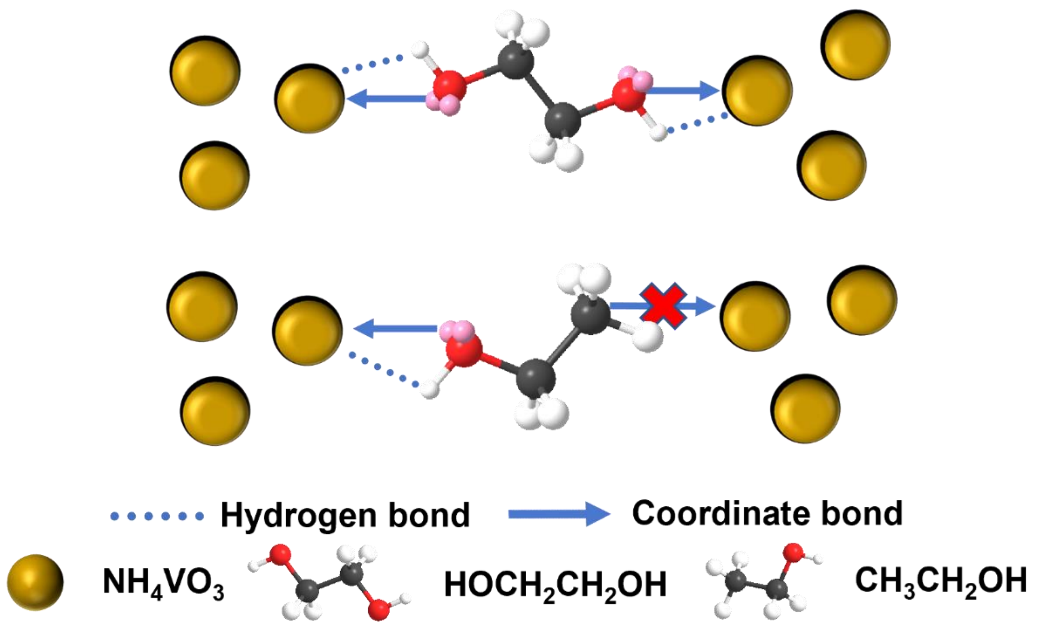

Figure S7. Schematic illustration of the chaining of ethylene glycol or ethanol with nanoparticles.

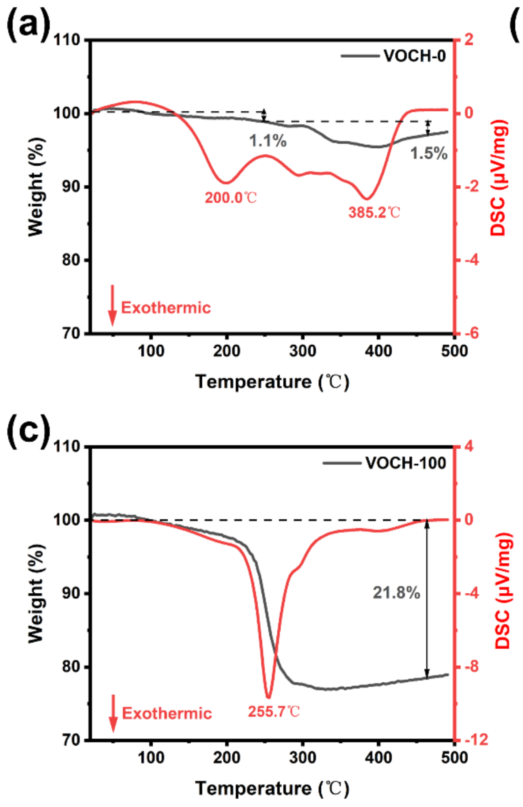

Figure S8. TGA/DSC profiles of (a) VOCH-0, (b) VOCH-20 and (c) VOCH-100 before calcination.

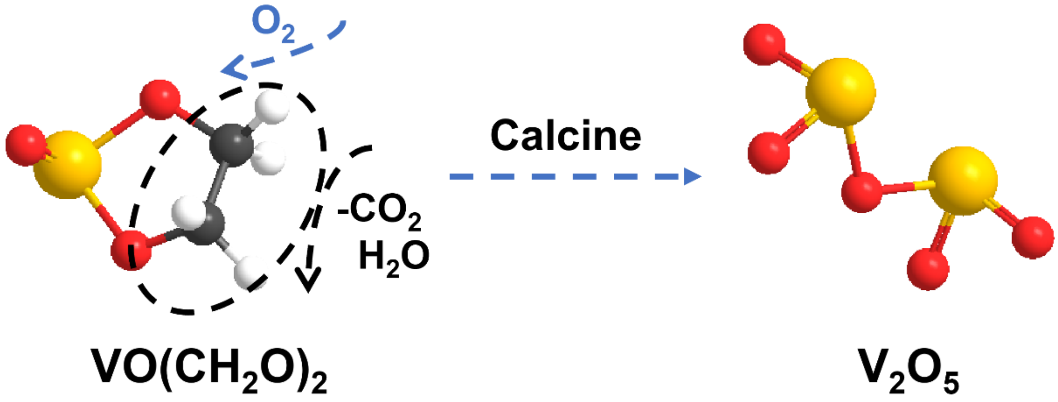

Figure S9. Schematic of V_2O_5 formation by calcination of VOCH precursors.

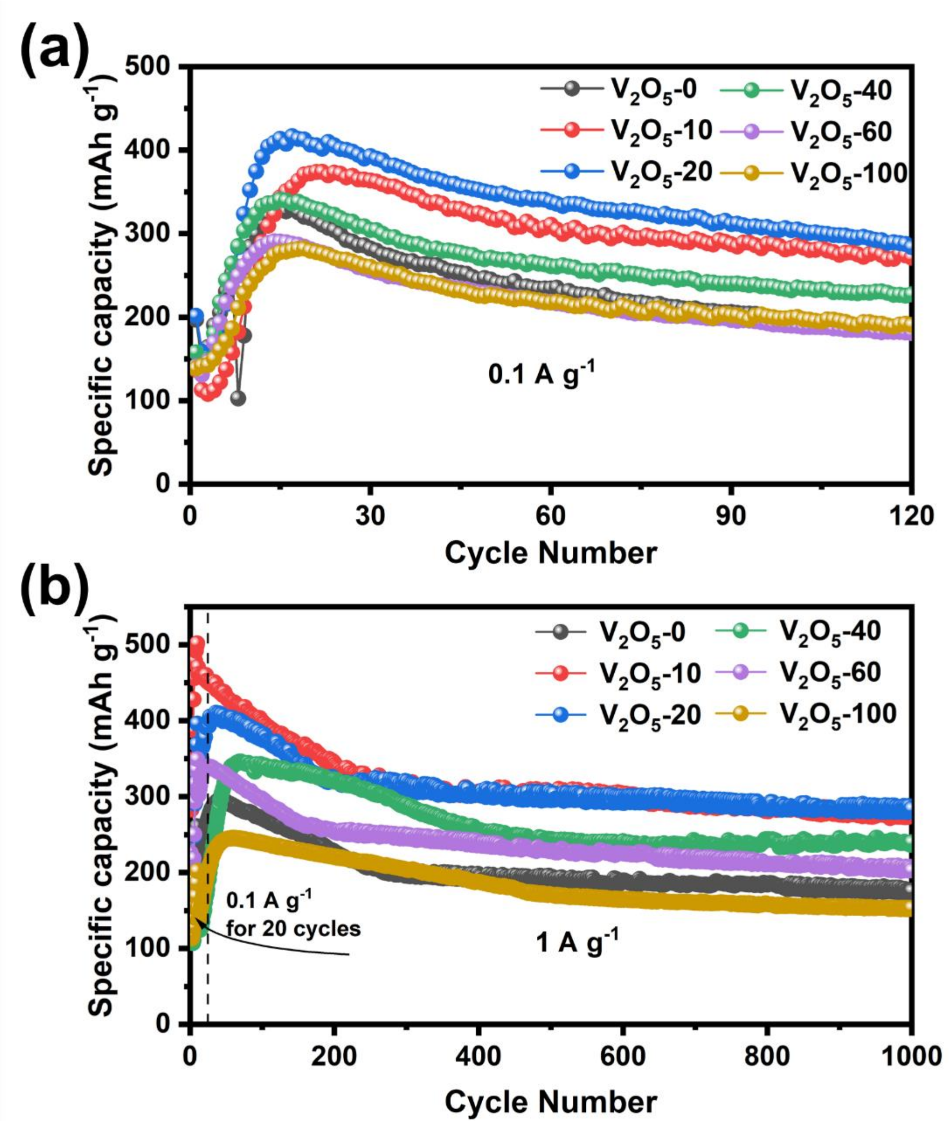

Figure S10. Cycle performance of different V_2O_5 electrodes at (a) 0.1 A g^{-1}, (b) 1 A g^{-1}.

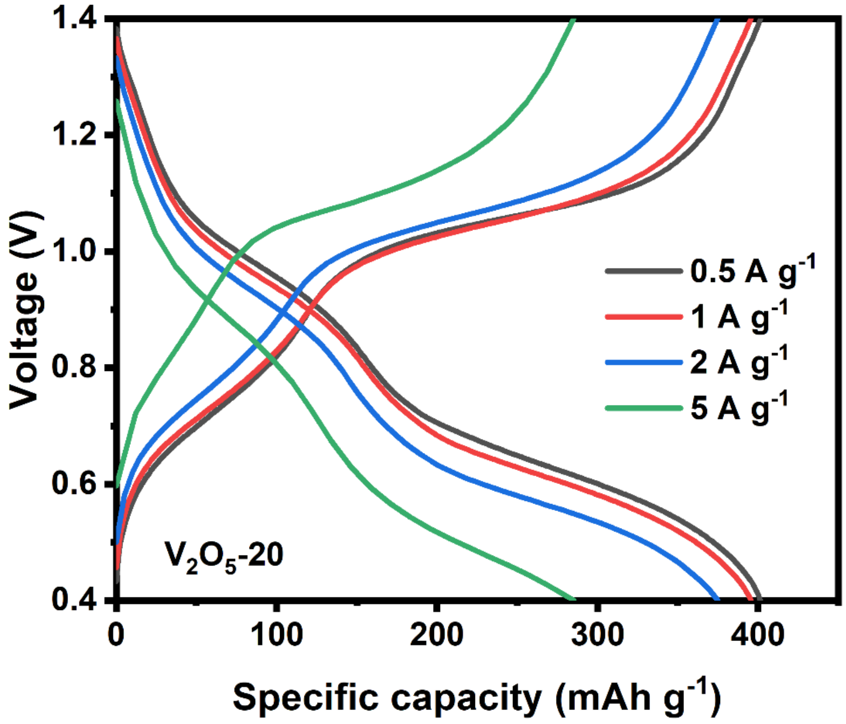

Figure S11. Specific capacity of V_2O_5-20 cathode at various current densities.

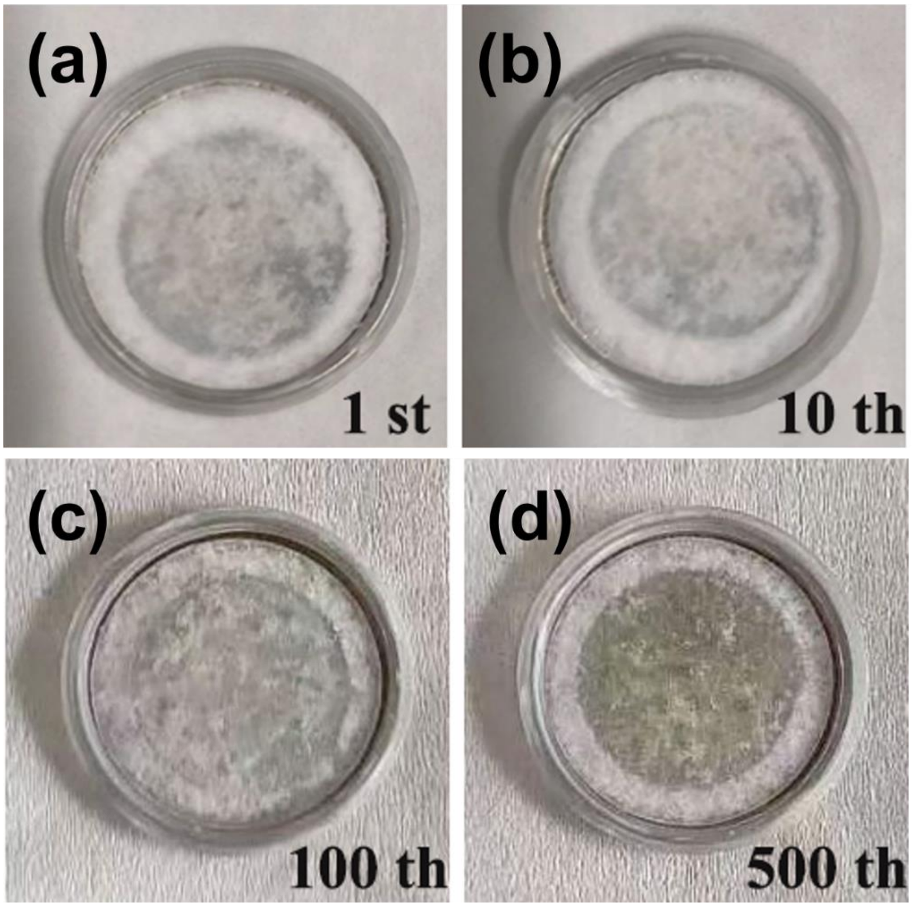

Figure S12. The pictures of battery separators after different recycle times: (a) 1st, (b) 10th, (c) 100th and (d) 500th.

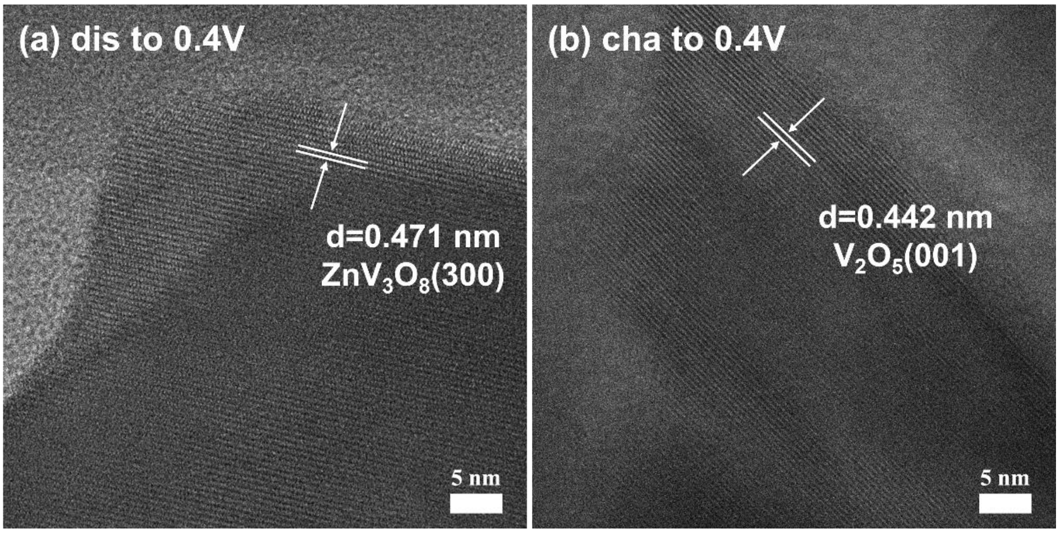

Figure S13. HRTEM images of V_2O_5-20 electrode (a) discharged to 0.4 V, (b) charged to 1.4 V.

Table S1. The BET surface area, pore volume and average pore size of V_2O_5-0 nanoparticles and V_2O_5-0 microspheres samples.

Sample	BET surface area (m^2g^{-1})	Pore volume (cm^3 g^{-1})	Average pore diameter(nm)
V_2O_5-0	6.8468	0.03272	10.1882
V_2O_5-20	9.4024	0.04763	37.0358

Table S2. A survey of V_2O_5-based electrode materials with three-dimensional structures for AZIBs.

Cathode material [Structure characteristic]	Electrolyte	Specific capacity	Cycling performance	Reference.
V_2O_5 (porous microspheres)	3M $Zn(CF_3SO_3)_2$	401mAh/g (0.1 A/g)	73% (1000) (2A/g)	[15]
V_2O_5 (hollow spheres)	Saturated $ZnSO_4$	280mAh/g (0.2 A/g)	82% (6200) (10A/g)	[22]
V_2O_5 (nanospheres)	3M $ZnSO_4$	327mAh/g (0.1 A/g)	69% (6000) (10A/g)	[41]
V_2O_5@CNTs (irregular spherical)	1 M $ZnSO_4$/1 Na_2SO_4	293mAh/g (0.3 A/g)	72% (6000) (5A/g)	[42]
VO_2 (hollow nanospheres)	3M $Zn(CF_3SO_3)_2$	440mAh/g (0.1 A/g)	47% (860) (1A/g)	[43]
